# Enhanced delivery of a low dose of aducanumab via FUS in 5×FAD mice, an AD model

**DOI:** 10.1186/s40035-022-00333-x

**Published:** 2022-12-27

**Authors:** Chanho Kong, Eun-Jeong Yang, Jaewoo Shin, Junwon Park, Si-Hyun Kim, Seong-Wook Park, Won Seok Chang, Chang-Han Lee, Hyunju Kim, Hye-Sun Kim, Jin Woo Chang

**Affiliations:** 1 Department of Neurosurgery, Yonsei University College of Medicine, 50 Yonsei-Ro, Seodaemun-Gu, Seoul, Republic of Korea; 2 Department of Pharmacology, College of Medicine, Seoul National University, 103 Daehakro, Jongro-Gu, Seoul, Republic of Korea; 3 Neuroscience Research Center, College of Medicine, Seoul National University, 103 Daehakro, Jongro-Gu, Seoul, Republic of Korea; 4 Department of Biomedical Sciences, College of Medicine, Seoul National University, 103 Daehakro, Jongro-Gu, Seoul, Republic of Korea; 5 Bundang Hospital, Seoul National University College of Medicine, Bundang-Gu, Sungnam, Republic of Korea

**Keywords:** Aducanumab, Alzheimer’s disease, Focused ultrasound, Transcriptome profiling

## Abstract

**Background:**

Aducanumab (Adu), which is a human IgG1 monoclonal antibody that targets oligomer and fibril forms of beta-amyloid, has been reported to reduce amyloid pathology and improve impaired cognition after administration of a high dose (10 mg/kg) of the drug in Alzheimer’s disease (AD) clinical trials. The purpose of this study was to investigate the effects of a lower dose of Adu (3 mg/kg) with enhanced delivery via focused ultrasound (FUS) in an AD mouse model.

**Methods:**

The FUS with microbubbles opened the blood–brain barrier (BBB) of the hippocampus for the delivery of Adu. The combined therapy of FUS and Adu was performed three times in total and each treatment was performed biweekly. Y-maze test, Brdu labeling, and immunohistochemical experimental methods were employed in this study. In addition, RNA sequencing and ingenuity pathway analysis were employed to investigate gene expression profiles in the hippocampi of experimental animals.

**Results:**

The FUS-mediated BBB opening markedly increased the delivery of Adu into the brain by approximately 8.1 times in the brains. The combined treatment induced significantly less cognitive decline and decreased the level of amyloid plaques in the hippocampi of the 5×FAD mice compared with Adu or FUS alone. Combined treatment with FUS and Adu activated phagocytic microglia and increased the number of astrocytes associated with amyloid plaques in the hippocampi of 5×FAD mice. Furthermore, RNA sequencing identified that 4 enriched canonical pathways including phagosome formation, neuroinflammation signaling, CREB signaling and reelin signaling were altered in the hippocami of 5×FAD mice receiving the combined treatment.

**Conclusion:**

In conclusion, the enhanced delivery of a low dose of Adu (3 mg/kg) via FUS decreases amyloid deposits and attenuates cognitive function deficits. FUS-mediated BBB opening increases adult hippocampal neurogenesis as well as drug delivery. We present an AD treatment strategy through the synergistic effect of the combined therapy of FUS and Adu.

**Supplementary Information:**

The online version contains supplementary material available at 10.1186/s40035-022-00333-x.

## Introduction

Alzheimer's disease (AD) is the most common neurodegenerative disease where cognitive functions, including memory, progressively deteriorate. Pathological features of AD include extracellular beta-amyloid (Aβ) plaques and intracellular neurofibrillary tangles, leading to neurodegeneration and neuronal cell death. There have long been only four FDA-approved treatments for AD, including donepezil, rivastigmine, and galantamine, which alleviate symptoms by inhibiting acetylcholinesterase activity, and memantine which is a partial NMDA antagonist [1]. According to the recently reported AD drug development pipeline and especially after the United States Food & Drug Administration (FDA) approved aducanumab (Adu), the recent treatment development tends to be progressing more toward the fundamental treatment of the disease rather than merely focusing on symptom relief [2, 3].

Adu, a human IgG1 antibody designed for targeting aggregated oligomer and fibril forms of Aβ, was approved by the FDA in 2021 [4] and is the first disease-modifying drug being used to slow the progression of AD and treat patients with mild cognitive impairment or the mild dementia stage of disease [5]. In a clinical study, the accumulation of Aβ in the hippocampus was decreased by Adu in a dose-dependent manner (3–10 mg/kg) [1]. Consistent with the aforementioned outcome of a clinical trial, an animal study using an AD mouse model (Tg2576) showed that Aβ was significantly reduced in the group given a dose of 10 mg/kg or higher (30 mg/kg) [5]. Acting as a double-edged sword, the blood-brain barrier (BBB) is essential for maintaining brain homeostasis. However, its function has long been a challenge in regard to applying potentially effective therapeutic agents, as BBB prevents approximately 98% of drug compounds with high molecular weight such as antibodies from penetrating the parenchyma [6]. Concentrating the acoustic pressure on the target area in the brain causes the cavitation effect of circulating microbubbles and temporarily opens the BBB [7]. Focused ultrasound (FUS) with microbubbles causes reversible opening of the BBB to both small and large molecules [8]. There are studies on the delivery of various therapeutic agents, such as chemotherapeutics [9–11] and cells [12–14]. Furthermore, multiple pieces of evidence have reported that cerebral Aβ levels and plaque burden are reduced by opening the BBB only without delivering therapeutic agents in AD mouse models [15–17]. Previously, we and other research groups reported that BBB opening by FUS increased adult hippocampal neurogenesis in rodents, which implies that FUS has therapeutic potential as an effective therapeutic strategy for AD [18–20].

Even though Adu has been approved by the FDA via an accelerated approval program, there may still be a challenging task in clinical phase IV. A previous study reported that only approximately 1.3% of Adu can reach the brain due to its large size by systemic administration. In addition, a high concentration (10–60 mg/kg) of Adu could not be used because of its side effects such as amyloid-related imaging abnormalities including edema (ARIA-E) or microhemorrhage/superficial siderosis (ARIA-H) [5]. In this study, we investigated the effects of a lower dose of Adu (3 mg/kg) with FUS in an AD mouse model.

## Methods

### Animals

The 5 familial AD mutations (5×FAD) mouse is a transgenic mouse with five familial mutations observed from early-onset AD families. This mouse expresses high levels of both mutant human amyloid precursor protein (APP695) with Swedish mutation (K670N, M671L), London mutation (V717I), Florida mutation (I716V), and human presenilin 1 (PS1) with two mutations (M146L and L286V). 5×FAD mice were purchased from Jackson Laboratory (Sacramento, CA) and maintained by crossing hemizygous transgenic mice with B6SJL F1 mice. The transgenic mice were identified by polymerase chain reaction, and non-transgenic littermates served as wild type (WT). The main characteristics of 5×FAD mice are extracellular amyloid deposition and gliosis beginning around 2 months [21]. Also, its phenotype can be defined as spontaneous alternation in the Y maze in that the impairment in spatial working memory begins at approximately 4–5 months of age [21, 22]. Neuron loss has been observed in multiple brain regions in this model and begins at about 6 months of age [21, 23]. All mice were housed in groups of 2–5 per cage with ad libitum access to food and water, in a humidity- and temperature-controlled, specific pathogen-free environment (12 h light cycle; lights on at 8 AM) in the Institute for Experimental Animals of Seoul National University. All experiments were approved by the Animal Care Committee of Seoul National University (Approval Number: SNU-201005-2-1).

### Focused ultrasound

**Fig. 1:**
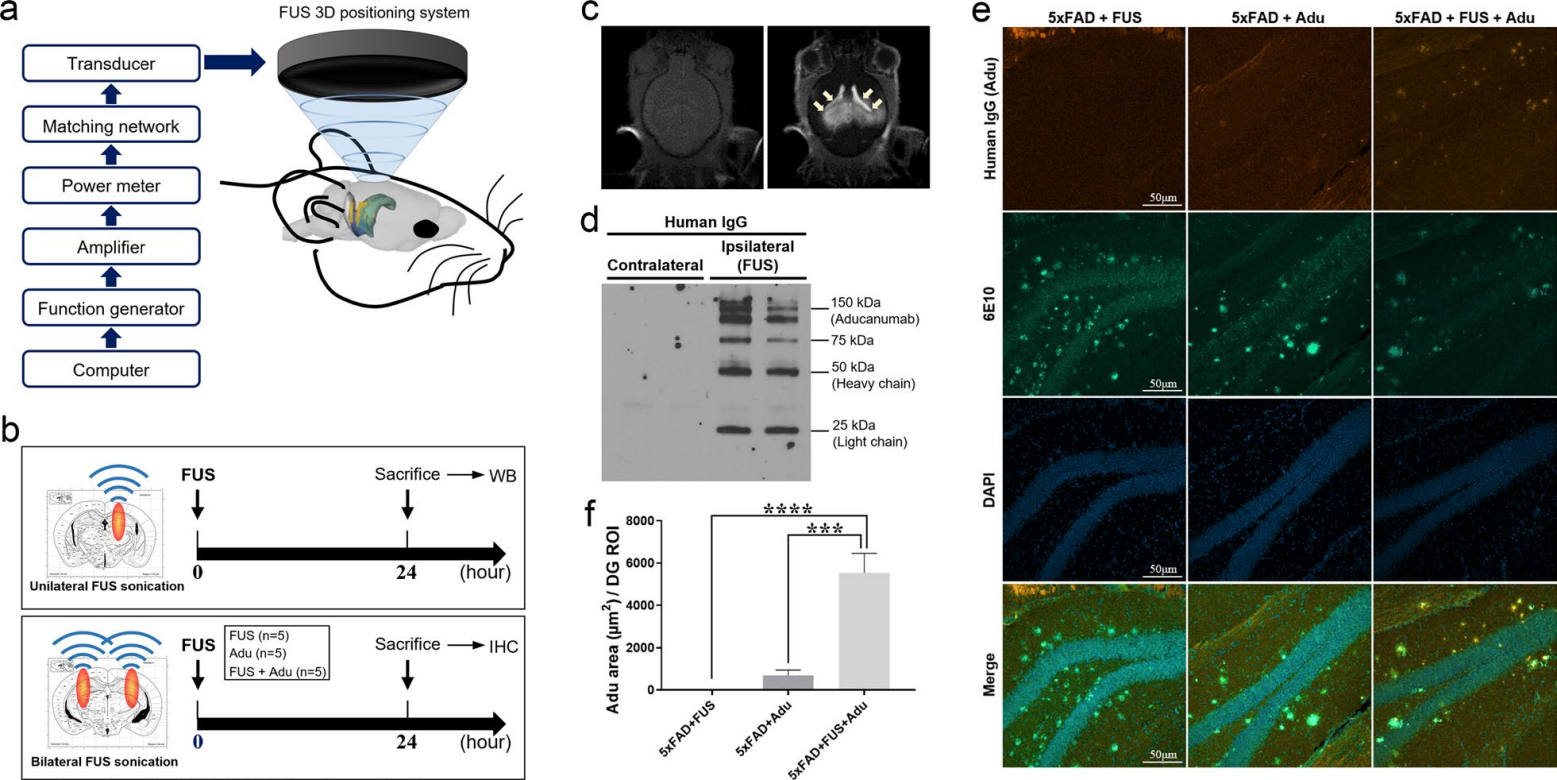
FUS-mediated BBB opening significantly increased the delivery of Adu in the brain. **a** A scheme of the FUS system set up for BBB opening in mice. **b** A schematic diagram for unilateral (upper panel) and bilateral (lower panel) FUS sonication. **c** Pre- and post-gadolinium T1-weighted images: FUS-mediated BBB opening was confirmed with MRI. **d** FUS was treated unilaterally to the brains of experimental animals. A representative image of Western blotting with an antibody against human IgG after Adu injection and unilateral FUS treatment. **e** Representative confocal images (20x) of human IgG (Aducanumab), Aβ stained with 6E10 antibody and DAPI in the dentate gyrus of the hippocampus. **f** A bar graph showing the levels of Adu assessed with human IgG antibody in the hippocampus. Data are presented as mean ± SEM. Statistical analyses were performed using one-way ANOVA, followed by Tukey’s post hoc analysis. (****P < 0.0001, ***P < 0.001, *n* = 5 mice for each group)

A 0.5 MHz single element focused transducer (H-107MR; SonicConcepts, Bothell, WA) was used. The diameter of the transducer was 51.7 mm and the radius of curvature was 63.2 mm. The transducer was used with a conical container that could be filled with degassed water to efficiently transfer acoustic energy. A waveform generator (33220A, Agilent, Palo Alto, CA) was connected to a 40 dB Radio Frequency Power Amplifier (210 L, ENI Inc., Rochester, NY) to drive the FUS transducer, and a power meter (E4419B, Agilent) was used to measure the input electrical power (Fig. 1a).

### Magnetic resonance imaging (MRI)

MRI was performed immediately following sonication with a Bruker 9.4 T 20 cm bore MRI system (Biospec 94/20 USR; Bruker, Ettlingen, Germany) and mouse head coil. A gadolinium-based MRI contrast agent, Gadobutrol (Gd; Gadovist; 0.2 ml/kg), was injected intravenously. MRI sequence parameters are summarized in Table 1.

**Table 1:** MRI sequences and parameters

	T1-weighted imaging
Echo	1
TR (ms)	500
TE (ms)	8.1
FA (deg)	90
NEX	5
FOV (cm)	2 × 2
Matrix	256 × 256

### Preparation of Adu

VH and VL sequences were identified in Biogen Idec’s patent submission for WO2014089500A1 and were cloned into human IgG2a and kappa pcDNA3.1 vectors (GenScript, NJ). Human Adu was produced using the Expi293 expression system and purified using protein A/G microbeads (Thermo Fisher Scientific, MA).

### Preparation of aggregated Aβ peptide

Aggregated Aβ was prepared according to a previous study [24]. Briefly, 1 mg of lyophilized Aβ (Anaspec, AS-20276, CA, USA) was dissolved in hexafluoroisopropanol (HFIP) (Sigma-Aldrich, 105228, MO). The Aβ–HFIP solution (1 mM) was incubated at room temperature for 30 min. Then, Aβ was lyophilized by evaporating HFIP in a fume hood overnight. To remove remaining HFIP and moisture, Aβ was transferred to a HyperVAC-LITE (Hanil, HVC-2124, Gyeonggido, Korea) and dried for 1 h.

### Enzyme-linked immunosorbent assay (ELISA)

To determine the concentration of Adu in the serum, 16.13 ng of aggregated Aβ per well was coated to 96-well ELISA plates (Thermo Fisher Scientific, #439,454) overnight at 4 °C. Then, the plates were blocked with phosphate-buffered saline (PBS) supplemented with 3% BSA for 2 h at room temperature. Next, 50-fold diluted serum in PBS was added and incubated for 1 h at room temperature. After washing with PBS with 0.05% (*v*/*v*) tween20 (PBS-T), the bound serum antibodies were detected by an HRP-conjugated rabbit anti-human IgG (Invitrogen, #A18903, 1:500, MA). After washing with PBS-T four times, 50 μl of 3,3’,5,5’-tetramethylbenzidine (Thermo Fisher Scientific, #34028) was added per well as a chromogen substrate. The plate was kept at room temperature for 2 min 30 s, and the reaction was terminated by adding 50 μl of 2 M H_2_SO_4_. Absorbance at 450 nm was measured on an infinite M200 PRO (TECAN). The results are presented after subtracting the control value from 5×FAD-Sham mouse serum value. For the standard curve of Adu in the serum, 5×FAD-Sham mouse serum was used. Serially diluted Adu (100 nM, 50 nM, 25 nM, 12.5 nM, 6.25 nM, 3.125 nM) was added in 50-fold diluted 5×FAD-Sham mouse serum, and the concentrations were determined using the same method as above.

### Confirmation of Adu delivery by FUS-mediated BBB opening

A schematic diagram for unilateral or bilateral FUS sonication is shown in Fig. 1b. First, FUS was unilaterally sonicated into the hippocampus of the 5×FAD mice to confirm whether Adu was delivered into the brains of 5×FAD mice by FUS-mediated BBB opening. Then, 24 h later, the mice were sacrificed and the contralateral hemispheres were compared. Second, we quantified Adu to determine whether the combined treatment with FUS enhanced the delivery of Adu into the brain. The 5×FAD mice were divided into three groups: the 5×FAD + FUS, 5×FAD + Adu, and 5×FAD + FUS + Adu. The FUS was sonicated to the hippocampus bilaterally. Adu was injected intravenously immediately after FUS sonication, and the mice were sacrificed 24 h later.

### Combined treatment with FUS and Adu

**Fig. 2:**
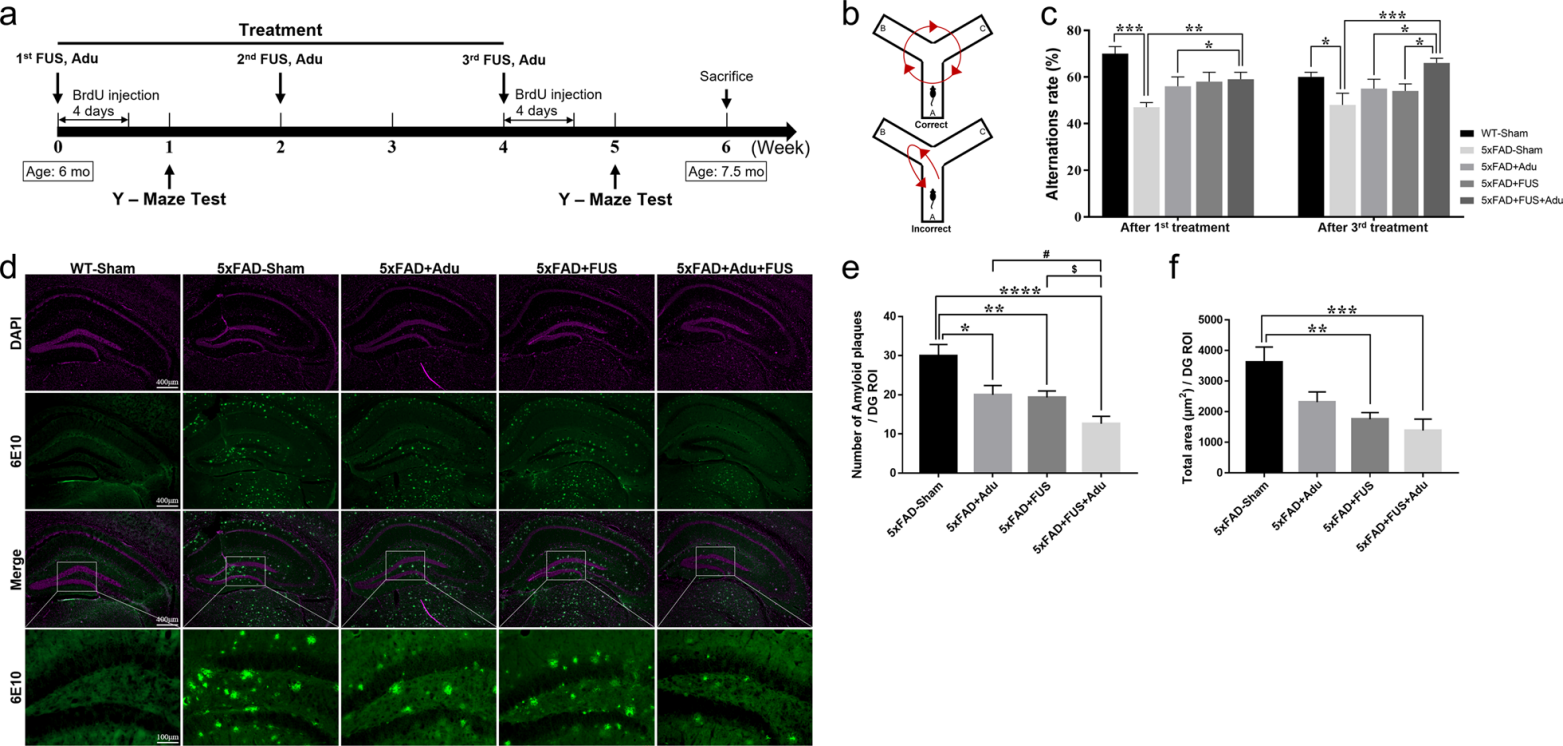
Treatment with FUS and Adu ameliorated cognitive impairment and levels of Aβ in the hippocampus. **a** Timeline of FUS and Adu treatment in 5×FAD mice. **b** Schematic illustration of the Y-maze spontaneous alternation test. **c** Alternation ratio in the Y-maze test at one week after the 1st treatment and at one week after the 3rd treatment. **d** Representative images (5×) of Aβ stained with 6E10 antibody and DAPI in the hippocampus. Bottom, representative images (20X) of Aβ stained with 6E10 antibody in the dentate gyrus of the hippocampus. **e** A bar graph showing the number of amyloid plaques in the dentate gyrus of the hippocampus (*P < 0.05, **P < 0.01, ***P < 0.001, and ****P < 0.0001 compared with 5×FAD-Sham mice, ANOVA followed by Tukey’s post hoc analysis, ^#^*P* < 0.05 compared with 5×FAD + Adu mice, ^$^*P* < 0.05 compared with 5×FAD + FUS mice by Student’s *t*-test, *n* = 11–15 mice for each group). **f** A bar graph showing the total area of amyloid plaques in the dentate gyrus of the hippocampus. Data are expressed as the means ± SEM. Statistical analyses were performed using one-way ANOVA, followed by Tukey’s post hoc analysis. (**P < 0.01, ***P < 0.001 compared with 5×FAD-Sham mice, *n* = 11–15 mice for each group)

All mice were divided into five groups: WT-Sham, 5×FAD-Sham, 5×FAD + Adu, 5×FAD + FUS and 5×FAD + FUS + Adu groups. For treatment, mice were anesthetized with 5% isoflurane in oxygen and animal heads were fixed on a stereotaxic frame (Narishige, Tokyo, Japan). Medical sterile ultrasound gel (ProGel-Dayo Medical Co, Seoul, South Korea) was used to fill the space between a coupling cone full of degassed water and the skull for energy transfer efficiency. The FUS was bilaterally targeted to four focal spots in the hippocampus. DEFINITY microbubbles (0.04 ml/kg; Lantheus Medical Imaging, North Billerica, MA) were injected intravenously 10 s before sonication. FUS (1-Hz burst repetition frequency, 10-ms bursts, 120 s in total, and average peak pressure 0.25 MPa) was started at the same time (Fig. 1a). Adu (3 mg/kg in saline) was injected intravenously at the end of FUS sonication. It was delivered three times in total and each treatment was performed every two weeks (Fig. 2a).

### Western blotting

Hippocampal lysates were resolved on a 10% SDS-PAGE gel and transferred to the nitrocellulose membrane, followed by blocking with 5% skim milk. The antibody used was anti-human IgG-HRP (Invitrogen, A18903, 1:2000, CA). The relative intensity of blots was quantified using ImageJ software.

### Spontaneous alternation Y-maze test

To investigate spatial working memory, spontaneous alternation in the Y-maze was investigated in experimental animals (Fig. 2b). Y-maze test was performed three times 1 week after each treatment (Fig. 2a). The alternation performance was tested using asymmetrical Y-maze, consisting of 3 equal arms (40 × 15 × 9 cm^3^), and constructed using black acrylic plastic. All mice were placed at the center of the Y-maze and allowed to explore freely for 8 min. All movements were recorded using a video camera and were analyzed to determine the alternation ratio by manually evaluating the number of triads containing entries into all three arms.

### 5-Bromo-2′-deoxyuridine (BrdU) labeling

BrdU was injected intraperitoneally twice a day for 4 days after treatment. To investigate the acute and chronic effects of treatment on cell proliferation, neuronal differentiation and cell survival in the hippocampus, we divided cells into two different time points of BrdU injection after the 1st and 3rd treatments. (Fig. 2a).

### Immunohistochemistry

Brains were fixed with 4% paraformaldehyde for 24 h, transferred to 30% sucrose for 3 days and stored at – 20 °C in cryoprotectant storage solution until use. Brains were cut into 30-µm coronal sections. Free-floating sections were washed in PBS and incubated in blocking solution (PBS, 5% normal goat serum, 0.2% Triton X-100) for 3 h at room temperature. Sections were incubated with primary antibodies in blocking solution overnight at 4 °C. The primary antibodies used were as follows: ZO-1 (Invitrogen, 61-7300, 1:1000, CA), BrdU (Abcam, ab6326, 1:250, Cambridge, UK), NeuN (Millipore, ABN78, 1:500, MA, USA), 6E10 (anti-Aβ aa 1–16 antibody, Biolegend, SIG39320, 1:500, CA), Iba-1 (Novus Biologicals, NB100-1028, 1:150, CO, USA or Wako, 19-19741, 1:150, VA), CD68 (Bio-Rad, MCA1957, 1:150, CA), and GFAP (Abcam, ab53554, 1:150, MA). After the primary immunoreaction, sections were incubated with Alexa 488 (Invitrogen, A11008, 1:500, CA)  or Alexa 594 (Abcam, A150156, 1:250, MA) conjugated secondary antibodies. For detection of Adu penetration into the brain, brain sections were incubated with anti-human IgG Alexa 555 (Invitrogen, A21433, 1:200, CA). Immunostaining of the sections was visualized with an LSM 700 confocal microscope (Carl Zeiss, Jena, Germany) or an Axio Imager M2 (Carl Zeiss) light microscope. Images were analyzed using ImageJ software (Version 1.52a, NIH, USA).

### Quantification of Aβ plaque-associated microglia and astrocytes

The quantification of Aβ plaque-associated microglia and astrocytes was performed using a previously reported method with minor modification [25]. Z-stacks (30 µm) were collected with 1 µm between each slice. Within 20 µm from the Aβ plaques, the number of Iba-1 or GFAP-positive cells was manually counted. Only those microglia or astrocytes whose nuclei were present in the Z-stack were included in the analysis. The size of the Aβ deposits was calculated from maximum projections of 6E10 immunoreactivity and subsequently used to determine the number of microglia or astrocytes surrounding the Aβ deposits of different sizes.

### RNA sequencing and ingenuity pathway analysis (IPA)

Three to four hippocampal samples per group were randomly selected for RNA sequencing analysis. Total RNA was extracted using a QIAGEN miRNeasy mini kit (Qiagen #217004). RNA (116 ng) was used to prepare RNA sequencing libraries using Illumina Stranded Total RNA Prep Ligation with Ribo-Zero Plus. The libraries were sequenced by an Illumina HiSeq 3000 sequencer at Yonsei Genome Center (Seoul, South Korea). The data preprocessing included a quality check by FastQC and, where needed, trimming of the adapter sequences by Trimmomatic v0.32. The raw reads were aligned to the mouse reference genome (mm10) using the HISAT2 splice-aware aligner. The transcripts per million were quantified using StringTie. Significantly differentially expressed genes (DEGs) were defined by adjusted *P* values (*q* values) less than 0.05. For the canonical pathway, upstream regulatory networks, and molecular networks of DEGs, we used the commercial QIAGEN Ingenuity^®^ Pathway Analysis (IPA^®^, QIAGEN Redwood City, www.qiagen.com/ingenuity) software. For the canonical pathway analysis, −log (*q* value) > 1.3 was taken as the threshold, a Z score > 1 was defined as the threshold of activation, and a Z score < − 1 was defined as the threshold of inhibition. For upstream regulators, –log (*q* value) > 1.3 was set as the threshold. The score was calculated by IPA for molecular networks, and significant changes in DEGs in the WT-Sham/5×FAD-Sham/5×FAD + FUS + Adu dataset were used for the comparison analysis.

### Statistical analysis

All data are expressed as the mean ± standard error of the mean. Data were calculated using a one-way analysis of variance (ANOVA) and Kruskal–Wallis test followed by a least significant different (LSD) and Tukey’s post hoc analysis. In addition, Student's *t*-test was also used. A value of *P* < 0.05 was considered statistically significant for all measures. All statistical analyses were performed using SPSS (Version 25, SPSS Inc., Chicago, IL) and GraphPad Prism 8 software (GraphPad Software Inc., San Diego, CA).

## Results

### FUS markedly improved the delivery of Adu into the targeted region of the brain, and Adu specifically bound to amyloid plaques in the hippocampi of 6–7 month-old 5×FAD mice

First, we confirmed the safety of FUS sonication in experimental animals. Previously, several groups, including our own, have demonstrated the safety of FUS [26, 27]. Here, we investigated the expression level of ZO-1 (Zonula occludens-1), which is a tight junction-associated protein reflecting the intactness of BBB, to confirm the safety of FUS in our model. The protein level of ZO-1 did not significantly differ among the WT-Sham, 5×FAD-Sham, 5×FAD + Adu, 5×FAD + FUS and 5×FAD + FUS + Adu groups 24 h after FUS sonication (Additional file 1: Fig. S1), indicating that there was no significant difference between the WT and 5×FAD or FUS sonication groups. Then, we investigated whether FUS with microbubbles can specifically open the BBB in targeted brain regions. After FUS sonication, we first acquired MR images without gadolinium and then performed MRI again with gadolinium to confirm whether the BBB is properly open. As shown in Fig. 1c, BBB opening in the hippocampus was confirmed through contrast-enhanced MRI. Then, to measure the increased delivery of Adu into the hippocampus after BBB opening by FUS treatment, we analyzed the hippocampal regions. FUS was given unilaterally (only to one hemisphere) to the hippocampus of experimental animals (Fig. 1b). A larger amount of intravenously administered Adu was detected in the ipsilateral region but not in the contralateral region (Fig. 1d).

We also quantified the amount of Adu in the brains 24 h after the treatment to investigate whether the combined treatment with bilateral FUS enhanced the delivery of Adu into the brains by immunohistochemistry. The FUS + Adu group markedly increased the delivery of Adu at 24 h compared with the Adu only group by approximately 8.1 times (Adu: 683 ± 259/dentate gyrus region of interest (ROI), FUS + Adu; 5541 ± 92 µm^2^/dentate gyrus ROI) (Fig. 1e and f). To confirm the specific binding of Adu with amyloid deposits in the hippocampus, co-staining with 6E10 and Adu antibodies was assessed. The colocalization of amyloid plaques with Adu was observed in the combined treatment group (Fig. 1e).

In addition, dose-dependent brain delivery of Adu by FUS was examined 24 h after FUS sonication in 6-month-old 5×FAD mice by performing Western blotting. A dose-dependent increase in the amount of Adu of the brain, as assessed with human IgG, was observed. In both the 3 and 10 mg/kg Adu without FUS groups, very little Adu was detected by Western blotting (Additional file 1: Fig. S2). Based on these results, the delivery of Adu (3 and 10 mg/kg) with FUS was significantly higher than the delivery of Adu without FUS, supporting our hypothesis that a lower dose of Adu (3 mg/kg) can exert therapeutic effects when applied with FUS sonication. The serum concentration of Adu was also investigated by ELISA assay with aggregated Aβ peptides at 1 and 24 h after intravenous injection with or without FUS sonication in 6-month-old 5×FAD mice. As expected, the 5×FAD + FUS + Adu group showed a significantly lower serum Adu concentration than the 5×FAD + Adu group. At a low dose (3 mg/kg), the 5×FAD + FUS + Adu group showed an approximately 33.5% lower Adu concentration than the 5×FAD + Adu after 1 h, and the 5×FAD + FUS + Adu group showed an approximately 50.5% lower Adu concentration than the 5×FAD + Adu after 24 h. Similarly, at a high dose (10 mg/kg), the 5×FAD + FUS + Adu group showed an approximately 12.9% lower Adu concentration than the 5×FAD + Adu after 1 h, and the 5×FAD + FUS + Adu group showed an approximately 38.8% lower Adu concentration than the 5×FAD + Adu after 24 h (Additional file 1: Fig. S3). These results are correlated with the brain results.

### Combined treatment with FUS and low dose of Adu (3 mg/kg) ameliorated cognitive impairments and significantly reduced the levels of amyloid plaques in the dentate gyrus of the hippocampus of 6 to 7-month-old 5×FAD mice

To investigate the effect of combined treatment with FUS and Adu on cognitive impairment in 5×FAD mice, spatial learning memory was assessed using the Y-maze test. After the 1st treatment, the combined treatment group with FUS and Adu featured a significantly increased alternation ratio compared with 5×FAD-Sham mice and 5×FAD + Adu mice (Fig. 2c, WT-Sham: 69.7 ± 2.5%, 5×FAD-Sham: 46.7 ± 2.1%, 5×FAD + Adu: 55.9 ± 3.6%, 5×FAD + FUS: 58.1 ± 4.1%, 5×FAD + FUS + Adu: 59.1 ± 2.6%). After the 3rd treatment, the combined treatment group exhibited a higher alternation ratio than the 5×FAD-Sham mice, the 5×FAD + Adu mice, and the 5×FAD + FUS mice (Fig. 2c, WT-Sham: 60.6 ± 2.1%, 5×FAD-Sham: 47.8 ± 4.5%, 5×FAD + Adu: 54.6 ± 3.6%, 5×FAD + FUS: 53.8 ± 3.8%, 5×FAD + FUS + Adu: 65.8 ± 2.0%). The combined treatment with FUS and Adu was significantly effective in alleviating the cognitive dysfunction assessed with the Y-maze test than the treatment with Adu or FUS alone. The combined treatment with FUS and Adu contributed to significantly improved cognitive impairment in the 5×FAD mice. To measure the therapeutic effects of combined treatment with FUS and Adu, we analyzed the accumulation of amyloid plaques in the hippocampus through immunostaining with 6E10 antibody. In the brains of WT-Sham mice, amyloid plaques were not observed at this age (7.5 months old, Fig. 2d).

All groups showed a significantly reduced number of amyloid plaques in the dentate gyrus of the hippocampus compared with 5×FAD-Sham; in particular, the decrease was remarkably reduced in the 5×FAD + FUS + Adu group (Fig. 2e, 5×FAD-Sham: 29.9 ± 2.9, 5×FAD + Adu: 20.0 ± 2.3, 5×FAD + FUS: 19.3 ± 1.6 and 5×FAD + FUS + Adu: 12.6 ± 1.9/dentate gyrus ROI). Additionally, areas of decreased Aβ were measured and compared. 5×FAD + FUS and 5×FAD + FUS + Adu groups showed a significantly reduced total area of amyloid plaques in the dentate gyrus of the hippocampus compared with 5×FAD-Sham group (Fig. 2f, 5×FAD-Sham: 3614 ± 498, 5×FAD + Adu: 2316 ± 331, 5×FAD + FUS: 1755 ± 218, 5×FAD + FUS + Adu: 1385 ± 374.3/dentate gyrus ROI). Thus, repeated combined treatment with FUS and Adu contributed to significantly improved cognitive impairment in the 5×FAD mice. Taken together, the combined treatment of Adu with FUS significantly ameliorated the amyloid plaque load in the hippocampi of 5×FAD mice compared with Adu or FUS alone.

### Combined treatment with FUS and Adu induced activation of phagocytic microglia and increased astrocytes associated with amyloid plaques in the hippocampi of 5×FAD mice

**Fig. 3:**
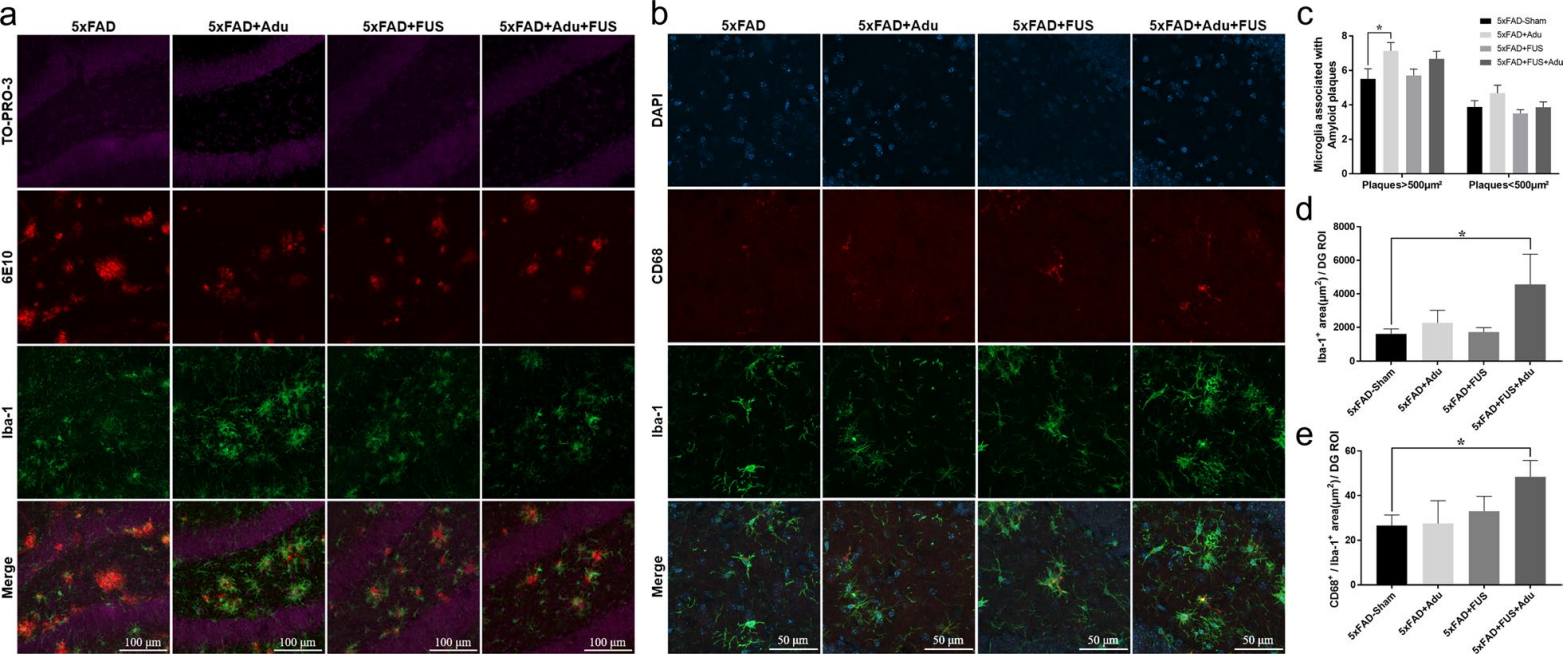
Treatment of FUS and Adu increased the number of activated phagocytic microglia associated with amyloid plaques in the hippocampus. **a** Representative images showing Iba-1-positive cells (green) surrounding Aβ plaques (red) in the dentate gyrus of the hippocampus. **b** Representative images of CD68 (red) and Iba-1(green) contained in the dentate gyrus of the hippocampus. **c** A bar graph showing the number of Iba1^+^microglia within 20 µm from Aβ plaques that were larger than 500 µm^2^ or smaller than 500 µm^2^. **d** A bar graph showing the total area of microglia. **e** A bar graph showing the phagosome area (CD68^+^/Iba1^+^) in microglia. Data are expressed as the means ± SEM. Statistical analyses were performed using one-way ANOVA, followed by Tukey’s post hoc analysis. (*P < 0.05 compared with 5×FAD-Sham mice, *n* = 3–4 mice for each group)

Microglia and astrocytes clear pathological deposits of molecules such as Aβ through phagocytosis and degradation in the central nervous system (CNS) [28]. To measure the localization of amyloid plaque-associated microglia, immunostaining of Iba-1, which is well known as a marker of microglia, was performed for the four experimental groups, including 5×FAD-Sham, 5×FAD + Adu, 5×FAD + FUS, and 5×FAD + FUS + Adu (Fig. 3a). The number of reactive microglia within 20 μm from amyloid plaques larger than 500 µm^2^ was significantly increased only in the 5×FAD + Adu group compared with the 5×FAD-Sham group (Fig. 3c, 5×FAD-Sham: 5.5 ± 0.60, 5×FAD + Adu: 6.8 ± 0.4, 5×FAD + FUS: 5.7 ± 0.3, 5×FAD + FUS + Adu: 6.6 ± 0.4/amyloid plaque). These data indicate that Adu treatment alone recruited microglia to large amyloid plaques. No significant difference was observed in the number of microglia within 20 μm  from amyloid plaques smaller than 500 µm^2^ in all groups (Fig. 3c, 5×FAD-Sham: 3.8 ± 0.3, 5×FAD + Adu: 4.6 ± 0.47, 5×FAD + FUS: 3.5 ± 0.2 and 5×FAD + FUS + Adu: 3.8 ± 0.3/amyloid plaque). Then, we examined the Iba-1^+^ microglial area and Iba-1^+^/CD68^+^ microglia to investigate whether the activation of microglia and potential phagocytic activity in microglia were altered in all groups. CD68, a marker associated with phagocytic activity, is increased in the AD brain [29]. The Iba-1^+^ area in the dentate gyrus of the hippocampus was increased in the combined treatment group compared with the 5×FAD-Sham group (Fig. 3b, d, and e; 5×FAD-Sham: 1600 ± 304 µm^2^, 5×FAD + Adu: 2264 ± 760 µm^2^, 5×FAD + FUS: 1716 ± 257 µm^2^ and 5×FAD + FUS + Adu: 4568 ± 1976 µm^2^/dentate gyrus ROI). The co-stained area with Iba-1 and CD68, which is a lysosome-associated membrane protein involved in phagocytosis and is used as a marker for macrophages and other mononuclear phagocytes, in the dentate gyrus of the hippocampus was significantly increased in the combined treatment group (Fig. 3e, 5×FAD-Sham: 26.6 ± 4.7 µm^2^, 5×FAD + Adu: 27.5 ± 10.1 µm^2^, 5×FAD + FUS: 33.0 ± 6.6 µm^2^, and 5×FAD + FUS + Adu: 48.4 ± 7.3 µm^2^).

**Fig. 4:**
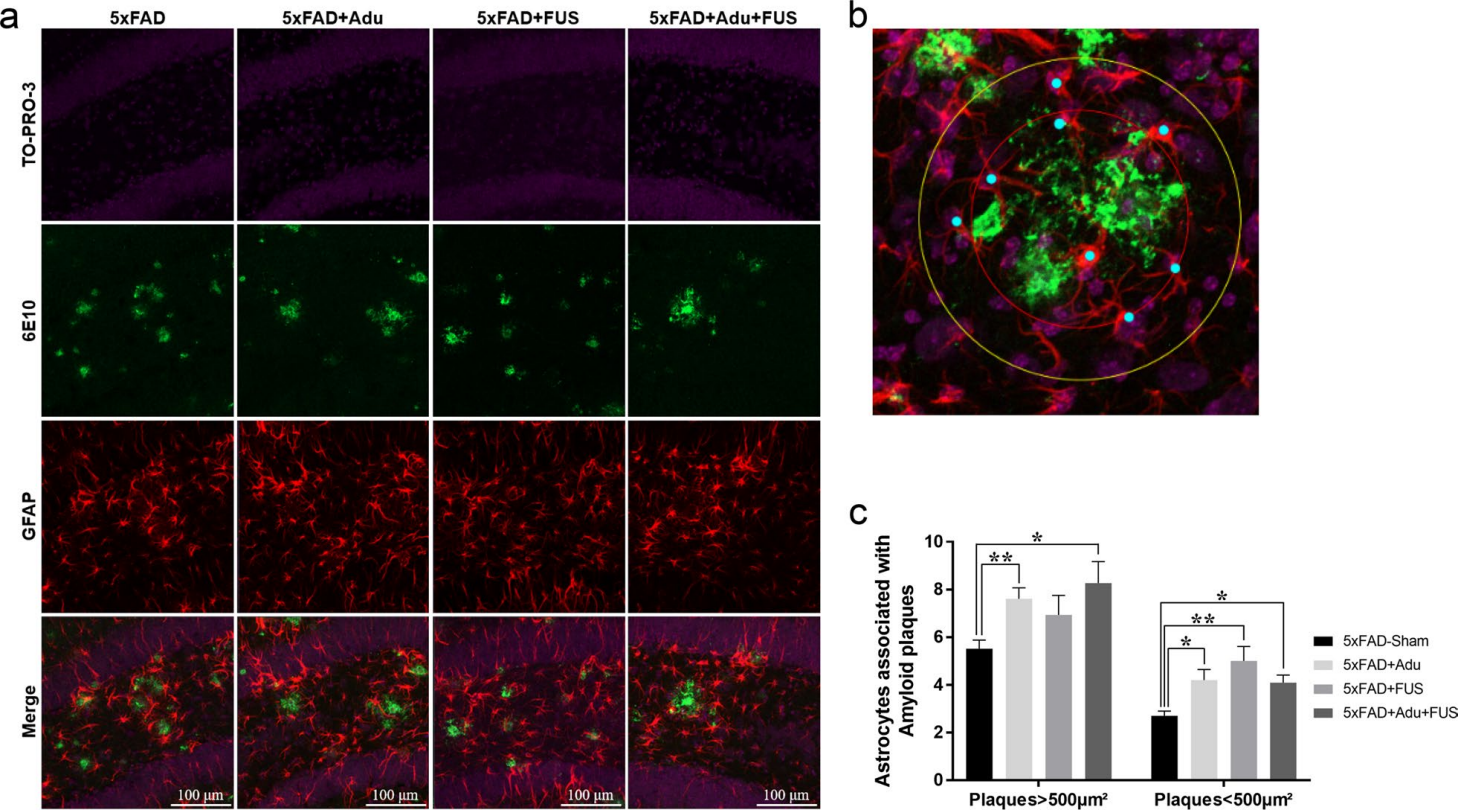
Treatment of FUS and Adu increased plaque-associated astrocytes in the hippocampus. **a** Representative images showing GFAP-positive cells (red) surrounding Aβ plaques (green) in the dentate gyrus of the hippocampus. **b** A representative higher-magnification image for the quantification of number of plaque-associated astrocytes. **c** A bar graph showing the number of astrocytes within 20 µm from Aβ plaques that were larger than 500 µm^2^ or smaller than 500 µm^2^. Data are expressed as the means ± SEM. Statistical analyses were performed using the Kruskal–Wallis test. (*P < 0.05 and **P < 0.01 compared with 5×FAD-Sham mice, *n* = 3–4 mice for each group)

Reactive astrocytes cluster around amyloid plaques, and the brain area occupied by these reactive astrocytes shows a significant increase in AD patients [30]. In addition, there are several lines of evidence that astrocytes can also eliminate amyloid plaques and neurons containing Aβ and enhance phagocytosis by microglia [31–33]. To investigate the number of amyloid plaque-associated astrocytes, we assessed the number of astrocytes within 20 μm from amyloid plaques. As shown in Fig. 4a, confocal images showed that reactive astrocytes were recruited to amyloid plaques in all groups. To describe how the number of astrocytes associated with plaques was measured, a higher-magnification image is shown in Fig. 4b. The recruitment of astrocytes surrounding amyloid plaques smaller than 500 μm^2^ was enhanced in the 5×FAD + Adu group, the 5×FAD + FUS group, and the combined treatment group (Fig. 4c, 5×FAD-Sham: 2.7 ± 0.2, 5×FAD + Adu: 4.20 ± 0.4, 5×FAD + FUS: 5.0 ± 0.6, 5×FAD + FUS + Adu: 4.1 ± 0.3/amyloid plaque). The recruitment of astrocytes was increased in 5×FAD mice treated with Adu alone and the combination treatment surrounding amyloid plaques larger than 500 μm^2^ (Fig. 4c, 5×FAD-Sham: 5.5 ± 0.3, 5×FAD + Adu: 7.6 ± 0.4, 5×FAD + FUS: 6.9 ± 0.8, and 5×FAD + FUS + Adu: 8.2 ± 0.9/amyloid plaques). These results suggest that the activation of microglia and the number of astrocytes associated with amyloid plaques were increased by the combined treatment with FUS and Adu.

### Combined treatment with FUS and Adu increased neurogenesis in the hippocampi of 5×FAD mice

**Fig. 5:**
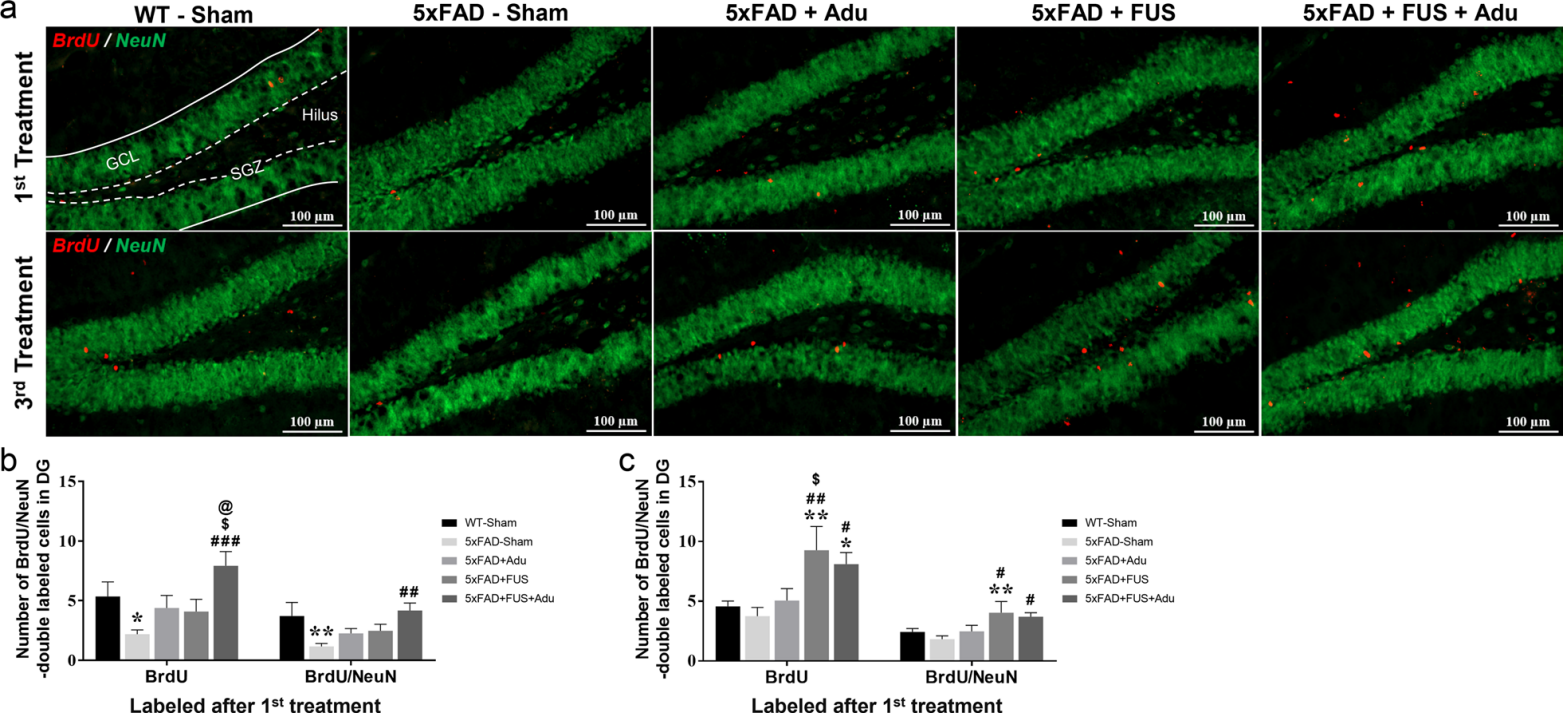
Treatment with FUS and Adu increased neurogenesis in the hippocampus. **a** Representative images showing immunofluorescence of neuronal nuclear marker (NeuN, green) and 5-bromo-2′-deoxyuridine (BrdU, red) in the dentate gyrus of the hippocampus. Scale bars: 100 µm. SGZ: subgranular zone, GCL; granular cell layer. **b** Bar graphs showing the number of BrdU- and BrdU/NeuN-positive cells after the 1st treatment. **c** Bar graphs showing the number of BrdU- and BrdU/NeuN-positive cells after the 3rd treatment. Data are expressed as the means ± SEM. Statistical analyses were performed using one-way ANOVA followed by a least significant difference post-hoc analysis. (*P < 0.05, and **P < 0.01, compared with WT-Sham mice, ^#^*P* < 0.05, ^##^*P* < 0.01, and ^###^*P* < 0.001 compared with 5×FAD-Sham mice, ^$^*P* < 0.05 compared with 5×FAD + Adu mice, ^@^*P* < 0.05 compared with 5×FAD + FUS mice, *n* = 5–10 for each group)

Adult hippocampal neurogenesis is normally active in neurologically normal subjects and decreases severely in patients with AD [34]. To determine whether repeated treatment affects the survival of labeled cells in the hippocampus, half of each group was injected with BrdU after the 1st treatment. The rest of mice was injected after the 3rd treatment to measure the cell proliferation and differentiation activity following the 3rd treatment. We quantified the cells stained with BrdU which is incorporated into dividing cells during the S-phase of the cell cycle and is a marker of newborn cells [35], and NeuN, a neuronal marker in the subgranular zone and granular cell layer of the dentate gyrus (Fig. 5a). The numbers of BrdU^+^ and BrdU^+^/NeuN^+^ cells after the 1st treatment and after the 3rd treatment were compared. Only the combined treatment group (6.5 ± 0.8) showed a significantly increased number of BrdU^+^ cells in the hippocampus, compared with the 5×FAD-Sham group (3.1 ± 0.8) after the 1st and 3rd treatments (Fig. 5b and c). The combined treatment group (3.5 ± 0.5) also exhibited a highly increased number of BrdU^+^/NeuN^+^ cells compared with 5×FAD-Sham (1.6 ± 0.2) after the 1st and 3rd treatments (Fig. 5b and c). Interestingly, when BrdU was injected after the 3rd treatment, the proliferation rate was significantly higher in the FUS alone group than in the combined treatment group. Overall, these results indicate that the combined treatment of FUS and Adu increases proliferation and survival of differentiated neural cells and induces remarkable neurogenesis in the hippocampus.

### DEGs associated with inflammation and synaptic plasticity pathways were revealed via transcriptome profiling in the hippocampi of 5×FAD mice treated with FUS and Adu

**Fig. 6:**
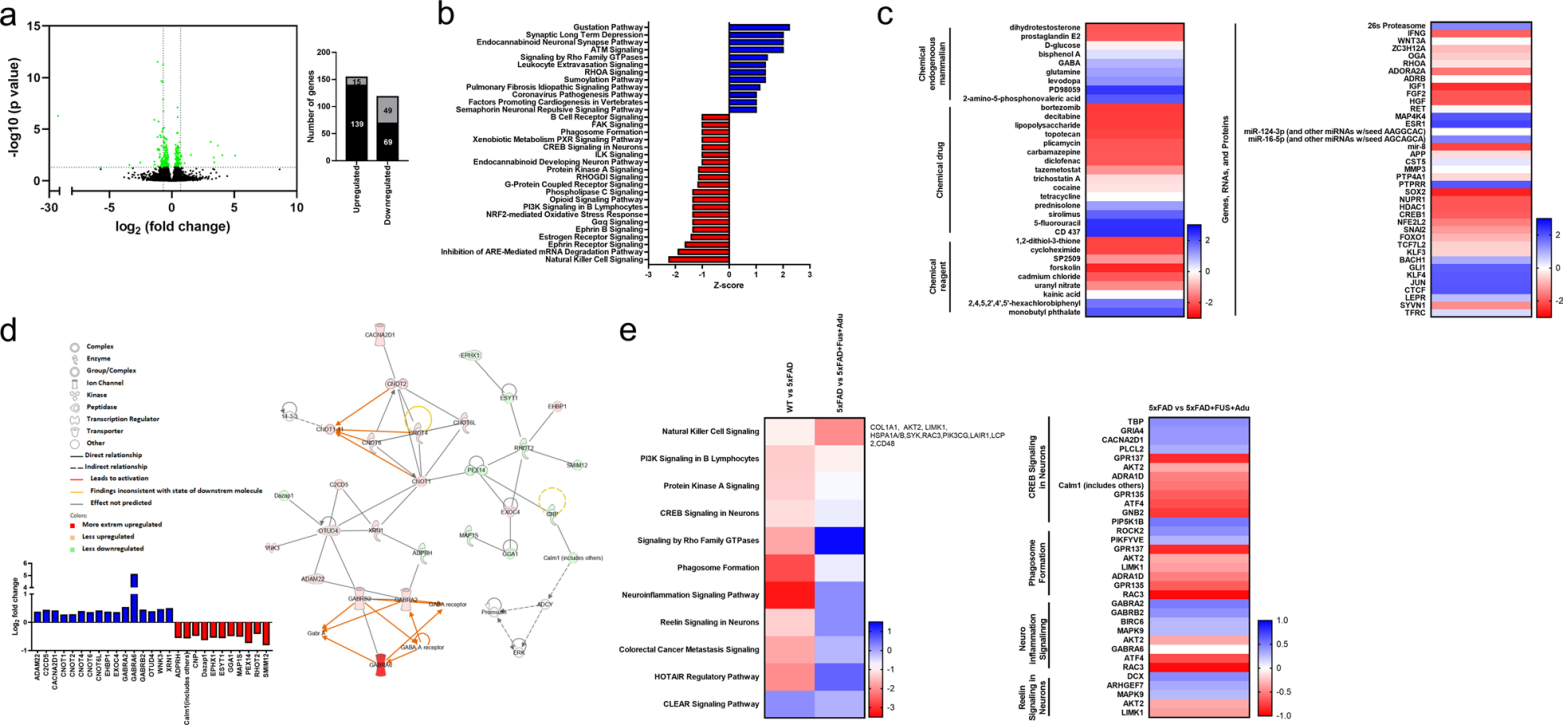
Treatment of FUS and Adu altered gene expression profiles in the hippocampus. **a** Left figure is a volcano plot. The x-axis represents the log_2_ conversion of the fold change (FC) values, and the y-axis represents the corrected significance level after base log_10_ conversion (*q* value). Green dots in the volcano plot and right graph indicate all DEGs that were found to differ significantly (*q* value < 0.05). The black bar in the right graph represents the number of genes with an absolute value of log_2_ FC greater than 0.7, and the gray bar represents the number of genes with an absolute value of log_2_ FC less than 0.7. **b** Canonical pathway analysis. Activated canonical pathway (blue bar) and inhibited canonical pathway (red bar) were identified (Z score > 1 or < − 1). **c** A heatmap of upstream regulators (Z score > 1 or < − 1). **d** A gene interaction network map (upper) and related DEGs’ log_2_ FC values (lower). **e** A heatmap of canonical pathways via comparison analysis (left) and four selected canonical pathways, including related molecules

To understand the underlying action mechanisms related to the recovery of cognitive impairments and neuropathological characteristics by treatment with FUS combined with Adu in 5×FAD mice, RNA sequencing was performed in the hippocampi of the experimental animal groups. As shown Fig. 6a, a total of 21,833 DEGs were identified and displayed as a volcano plot. Among these genes, 146 DEGs were up-regulated and 128 DEGs were down-regulated significantly (*q* < 0.05). Among the 274 annotated DEGs, a functional prediction analysis was performed using IPA software (Fig. 6b–e). For canonical pathway analysis, a total of 32 enriched canonical pathways were identified by applying absolute *z* scores greater than 1. Among these pathways, a total of 12 pathways and 20 pathways were activated and inhibited, respectively (Fig. 6b). Furthermore, a total of 276 upstream regulators which modulate DEGs were identified by applying the *p* value of overlap < 0.05 threshold. Among them, 30 molecules were predicted to be activated and 43 molecules were predicted to be inhibited (Fig. 6c). We also analyzed the network to show the interactions between molecules in the dataset (Fig. 6d). The highest ranked network which was sorted using the score values (score 49), was found to mainly affect ‘neurological disease, psychological disorder, organismal injury and abnormalities’, involving 27 molecules. We found that 16 DEGs (*ADAM22, C2CD5, CACNA2D1, CNOT1, CNOT2, CNOT4, CNOT6, CNOT6L, EHBP1, EXOC4, GABRA2, GABRA6, GABRB2, OTUD4, WNK3* and *RN1*) were up-regulated and 11 DEGs (*ADPRH, Calm1, CNP, Dazap1, EPHX1, ESYT1, GGA1, MAP1S, PEX14, RHOT2,* and *SMIM12*) were down-regulated in the network. To investigate the similarity, difference and trend between the WT-Sham and 5×FAD-Sham and between 5×FAD-Sham and 5×FAD + FUS + Adu datasets, a comparison analysis was performed using the comparison analysis function in IPA. As shown Fig. 6e, a total of 11 canonical pathways were found to be enriched in comparison analysis. In particular, four canonical pathways (phagosome formation, neuroinflammation signaling, CREB signaling in neurons and reelin signaling in neurons) were ameliorated in 5×FAD + FUS + Adu mice compared with 5×FAD mice. Taken together, our data revealed that combined treatment with FUS and Adu altered pathways in the hippocampus that are related to neuroflammation and neural activity.

## Discussion

The BBB hinders therapeutic agents from penetrating into the brain and becomes an obstacle to CNS disease treatment [36]. Previously, we reported several studies regarding BBB opening by FUS [20, 27]. The safety of FUS has already been verified, and it has been currently used in clinical trials [11, 37, 38].

A significant correlation between cognitive decline and brain amyloid plaque levels in the living brain evaluated using PET-CT scan has been reported [39]. Recently, the FDA approved Adu for the treatment of AD based on an evaluation of effects of the drug in clinical stages [5, 40]. However, the high dose of Adu (10–60 mg /kg) used in clinical studies induced ARIA-E in human clinical phases [41]. According to the study, patients who were treated with 10 mg/kg of Adu experienced ARIA-E with headache, confusion, dizziness and nausea; microhemorrhage; and superficial siderosis in clinical phase 3. Therefore, delivering an appropriate dose of Adu may be a key point for safety and effectiveness in AD.

In this study, we aimed to investigate whether the combined treatment with FUS and Adu improves the very low BBB penetration ratio of Adu caused by its large molecular weight (approximately 150 kDa) in systemic administration. We first confirmed that FUS safely opened the BBB (Fig. 1c). The Western blotting and immunofluorescence results demonstrated that the delivery of Adu was improved by FUS-mediated BBB opening (Fig. 1d–f, Additional file 1: Fig. S2). Additionally, FUS induced localized BBB opening and the opening was closed within 24 h (data not shown). These data imply that FUS is a potential and safe method to reversibly enhance BBB permeability and improve cerebral drug delivery.

Here, the impairments in cognitive function and the accumulation of amyloid plaques were ameliorated at a low dose of Adu (3 mg/kg) with FUS in 5×FAD mice (Fig. 2). While the combined treatment group only showed significant restoration of cognitive impairment, spontaneous alternation Y-maze test did not show any significant difference between the FUS alone or Adu alone group and 5×FAD-Sham group. Notably, the combined treatment resulted in a marked improvement in cognitive impairment after the 3rd treatment (Fig. 2c). In addition, we also examined neuropathological changes, especially the amyloid plaque levels in the hippocampus, after treatment in 5×FAD mice. The number of amyloid plaques was decreased in the hippocampi of the 5×FAD + Adu, 5×FAD + FUS, and 5×FAD + FUS + Adu mice. In addition, the total area of the amyloid plaques was reduced in the hippocampi of the 5×FAD + FUS and 5×FAD + FUS + Adu mice (Fig. 2d-f). A previous report by others focused on the effects of a scanning ultrasound on the delivery of Adu into the brain and demonstrated that in APP23 mice, both the Adu only and scanning ultrasound only groups reduced the total plaque area in the hippocampus with no additive effect observed with the combination treatment of scanning ultrasound and Adu [15, 17, 42].

In our study, somewhat different aspects of FUS was investigated. Although the effects of FUS on BBB opening and on neurogenesis may have different mechanisms and different biological relevance to AD treatment, we expect their effects would be synergistic.

Even though the FDA approved the use of Adu via an accelerated approval program, the action mechanism underlying the treatment effects of Adu in the brain is still poorly understood. To understand the underlying mechanisms of action, we investigated the changes in microglia, astrocytes, and neurons after treatment with Adu in 5×FAD mice. Microglia are the only immune cells resident in the CNS, constitute 5%–10% of total brain cells, and take up, phagocytose, and proteolyse both soluble and fibrillar forms of Aβ [43, 44]. Phagocytes such as microglia express Fc receptors (FcRs) on the cell surface and bind to the Fc region of antibodies. FcR activates phagocytosis, clearance of myelin debris and the inflammatory response [45, 46]. The Fc portion of Adu can bind to FcRs expressed in microglia and opsonize Aβ for phagocytosis by microglia [47]. Early reports found that microglia surround amyloid plaques in the brains of AD patients, and Aβ fibrils were found within the microglia [48]. In this study, treatment with Adu alone increased the recruitment of microglia to amyloid plaques. This might induce a decrease in the number of amyloid plaques as shown in Fig. 2d and e. However, the combined treatment did not affect the number of microglia surrounding the amyloid plaques (Fig. 3c). This finding indicates that the recruitment of microglia around the amyloid plaques was not changed. Interestingly, a quantitative assessment of the CD68^+^/Iba-1^+^ area revealed a significant increase in the 5×FAD + FUS + Adu group (Fig. 3e). Furthermore, we identified that the phagosome formation pathway (*PIP5K1B, ROCK2, PIKFYVE, GPR137, AKT2, LIMK1, ADRA1D, GPR135,* and *RAC3*) was activated in the combined treatment group using RNA sequencing and IPA (Fig. 6e). The activation of astrocytes, as demonstrated by increased GFAP expression, and amyloid deposition surrounded by activated astrocytes have a substantial impact on the AD state [49]. In the brains of AD patients and mouse models, there is a significant increase in GFAP immunoreactivities in plaque-associated astrocytes. Similar to the activation of microglia, reactive astrocytes phagocytose amyloid aggregates and dystrophic neurites and are involved in the inflammatory response to Aβ [33, 50]. Additionally, knockout of GFAP in an AD mouse model showed a twofold increase in amyloid plaque burden and twice the amounts of dystrophic neurites [51]. Astrocytes were reported to be activated and uptake more Aβ in the brains of MRI-guided FUS-treated mice [16]. Consistent with these results, we observed an increased number of plaque-associated astrocytes and a reduced number and size of amyloid plaques in hippocampi of the 5×FAD + Adu, 5×FAD + FUS, and 5×FAD + FUS + Adu mice (Figs. 2 and 4). Although treatment with Adu alone or FUS alone increased plaque-associated astrocytes, these increases were not sufficient to alleviate cognitive decline in the 5×FAD mice. Collectively, our data suggest that the combined treatment with FUS and Adu promotes glial phagocytosis (microglia and astrocytes) and clearance of Aβ, which may induce a reduction in Aβ deposition in the brains of the 5 × FAD mice. To elucidate the precise molecular mechanisms of phagocytosis associated with these pathways, more in-depth study is required in both in vitro and in vivo models.

The neurotoxicity of amyloidogenic proteins was demonstrated to affect long-term potentiation, plasticity, synaptic signaling, dendritic morphology, and cognition in a preclinical study. This toxic microenvironment decreases the survival and proliferation of newborn cells in the hippocampus [52–54]. Our results showed that the combined treatment decreased amyloid plaque formation as shown in Fig. 2.

Previous studies have reported that FUS-mediated BBB opening induces hippocampal neurogenesis [17, 18]. In this study, we investigated whether the combined treatment also induces neurogenesis and compared the effects of the combined treatment with FUS or Adu alone. The three administrations of combined treatment with FUS and Adu led to increased survival of newly proliferating and differentiated neuronal cells compared with FUS or Adu alone. Indeed, BrdU-labeled cells after the last combined treatment and FUS alone showed increased proliferation and neuronal differentiation (Fig. 5). Hippocampal neurogenesis plays a key role in long-term memory and cognitive function [55, 56]. Future research is needed to elucidate the difference between the results of the 1st and 3rd treatments. As neurogenesis is induced only when the BBB is opened, it is assumed that changes in the intravascular microenvironment or the components of the tight junction may have played a role in promoting neurogenesis. In addition, brain-derived neurotrophic factor (BDNF) is reported to be one of the most important factors in inducing neurogenesis, and there is a report that the FUS-mediated BBB opening increases the expression level of BDNF [20, 57]. Taken together, these results show that combined treatment enhances the survival of newborn cells, suggesting that Adu may potentially affect a part of the toxic microenvironment.

To understand the dynamic molecular processes induced by the combined treatment at the transcriptional level, transcriptome profiling was performed using RNA sequencing. We identified 32 canonical pathways based on significant DEGs that were submitted to IPA core analysis (Fig. 6a and b). To narrow the signaling pathways, a comparison analysis was conducted among multiple groups (WT vs 5×FAD and 5×FAD vs 5×FAD + FUS + Adu, Fig. 6e). We found four promising target canonical pathways, the neuroinflammation, phagosome formation, CREB and reelin signaling pathways. The combined treatment with FUS and Adu ameliorated neuroinflammation signaling which involved *GABRA2, GABRB2, BIRC6, MAPK9, AKT2, GABRA6, ATF4,* and *RAC3* and phagosome formation. These results may explain why microglia and astrocytes were activated by the combined treatment and may indicate which molecules are associated with neuroinflammation and phagocytosis (Figs. 3 and 4). Both CREB signaling (*TBP, GRIA4, CACNA2D1, PLCL2, GPR137, AKT2, ADRA1D, Calm1, GPR135, ATF4,* and *GNB2*) and reelin signaling (*DCX, ARHGEF7, MAPK9, AKT2*, and *LIMK1*) genes in neurons were proposed to be activated genes after combined treatment with FUS and Adu in 5×FAD (Fig. 6e). CREB signaling modulates synaptic plasticity by mediating the conversion of short- and long-term memory and has been involved in cognitive function [58]. Reelin signaling is also important in synaptic function, learning, and memory [59]. Thus, the comparison analysis supports the idea that cognitive impairment may be attenuated via these two pathways after combined treatment with FUS and Adu. Overall, transcriptome profiling suggests that gene sets related to an activated immune response (neuroinflammation and phagosome formation) in the brain and the inhibition of neuronal activity (CREB and reelin signaling) were reversed after combined treatment. Moreover, the IPA-derived gene network which provides direct and indirect relationships among DEGs and DEG regulators, suggests that the categories of neurological disease, psychological disorders, organismal injury, and abnormalities were scored highly after combined treatment with FUS and Adu. In particular, GABAergic pathway-associated genes (*GABRB2*, *GABRA2* and *GABRA6*) involved in this category were predicted in 5×FAD + FUS + Adu mice through network analysis (Fig. 6d). Several studies have reported the role of impaired function of GABA_A_ receptors by modulating neuronal activity in AD [50, 51]. Accordingly, these results may indicate that the GABAergic pathway also plays a role after combined treatment with FUS and Adu. To investigate the specific biological progress of Adu therapy in AD, further in-depth studies are needed to validate the potential key molecules or mechanisms identified in the current study.

## Conclusion

In conclusion, an effective treatment approach for AD is by improving cognitive function and reducing deposited amyloid plaques rather than alleviating symptoms and delaying progression. In this study, the enhanced delivery of a low dose of Adu (3 mg/kg) via FUS reduced amyloid deposits and restored spatial memory. In addition, it was proven to be effective in neuropathological changes such as enhanced phagocytosis and neurogenesis. Overall, this study provides insight into establishing a therapeutic strategy for the treatment of AD as well as other neurodegenerative diseases (Additional file 1: Fig. S4).

In this work, we demonstrated that FUS with microbubbles induced BBB opening and increased the delivery of Adu into the brain. In in vivo experiment, the combined treatment alleviated the pathology and improved cognitive function through glial stimulation and prevention of Aβ aggregation in the AD mouse model. In addition to achieving higher efficacy in the delivery and therapy, the combined treatment also showed safety in mice. This study suggests that the optimized delivery technology using FUS can be applied to other drugs for CNS diseases.

## Supplementary Information

**Additional file 1**. **Fig. S1**: The protein level of ZO-1 in 6-month-old WT-Sham, 5×FAD-Sham, 5×FAD+Adu, 5×FAD+FUS and 5×FAD+FUS+Adu mice. A representative blot of ZO-1 in the hippocampus is shown. **Fig. S2**: Brain delivery of Adu by FUS occurred in a dose-dependent manner and was examined 24 hours after FUS sonication in 6-month-old 5×FAD mice. A representative blot of Adu assessed with human IgG in the hippocampus is shown. **Fig. S3**: The Adu concentration in mouse serum after Adu treatment at two time points, 1 hour (white) and 24 hours (gray). A bar graph showing the levels of Adu determined using anti-human IgG antibody in serum. Data are presented as mean ± SEM. Statistical analyses were performed using one-way ANOVA, followed by Tukey’s post-hoc test. (*P < 0.05, **P < 0.01 compared with 5×FAD+Adu mice, *n* = 3 for each group). **Fig. S4**: Summary figure. Schematic diagram of the combined therapy of FUS and Adu. FUS can be used in conjunction with microbubbles to temporarily open the BBB, thereby allowing delivery of Adu into the brain. Due to the combined effect of FUS and Adu, neurogenesis and phagocytosis are increased and amyloid plaques are decreased.

## Data Availability

The datasets used and/or analyzed in the current study are available from the corresponding authors on reasonable request.

## Abbreviations

Aβ: Amyloid-β
AD: Alzheimer’s disease
Adu: Aducanumab
ARIA-E: Abnormalities including edema
BBB: Blood–brain barrier
BDNF: Brain-derived neurotrophic factor
BrdU: 5-Bromo-2′-deoxyuridine
CNS: Central nervous system
DEG: Differentially expressed gene
FcR: Fc receptor
FUS: Focused ultrasound
GABA: Gamma-aminobutyric acid
GCL: Granular cell layer
GFAP: Glial fibrillary acidic protein
IPA: Ingenuity pathway analysis
MRI: Magnetic resonance imaging
NeuN: Neuronal nuclei
PBS: Phosphate-buffered saline
RNA: Ribonucleic acid
ROI: Region of interest
SGZ: Subgranular zone
WT: Wild type
