# Supplementary figures and images for: Enhanced delivery of a low dose of aducanumab via FUS in 5×FAD mice, an AD model
Source: Transl Neurodegener. 2022 Dec 27;11:57. doi: 10.1186/s40035-022-00333-x (PMC9793531; doi:10.1186/s40035-022-00333-x)

## Slide 1
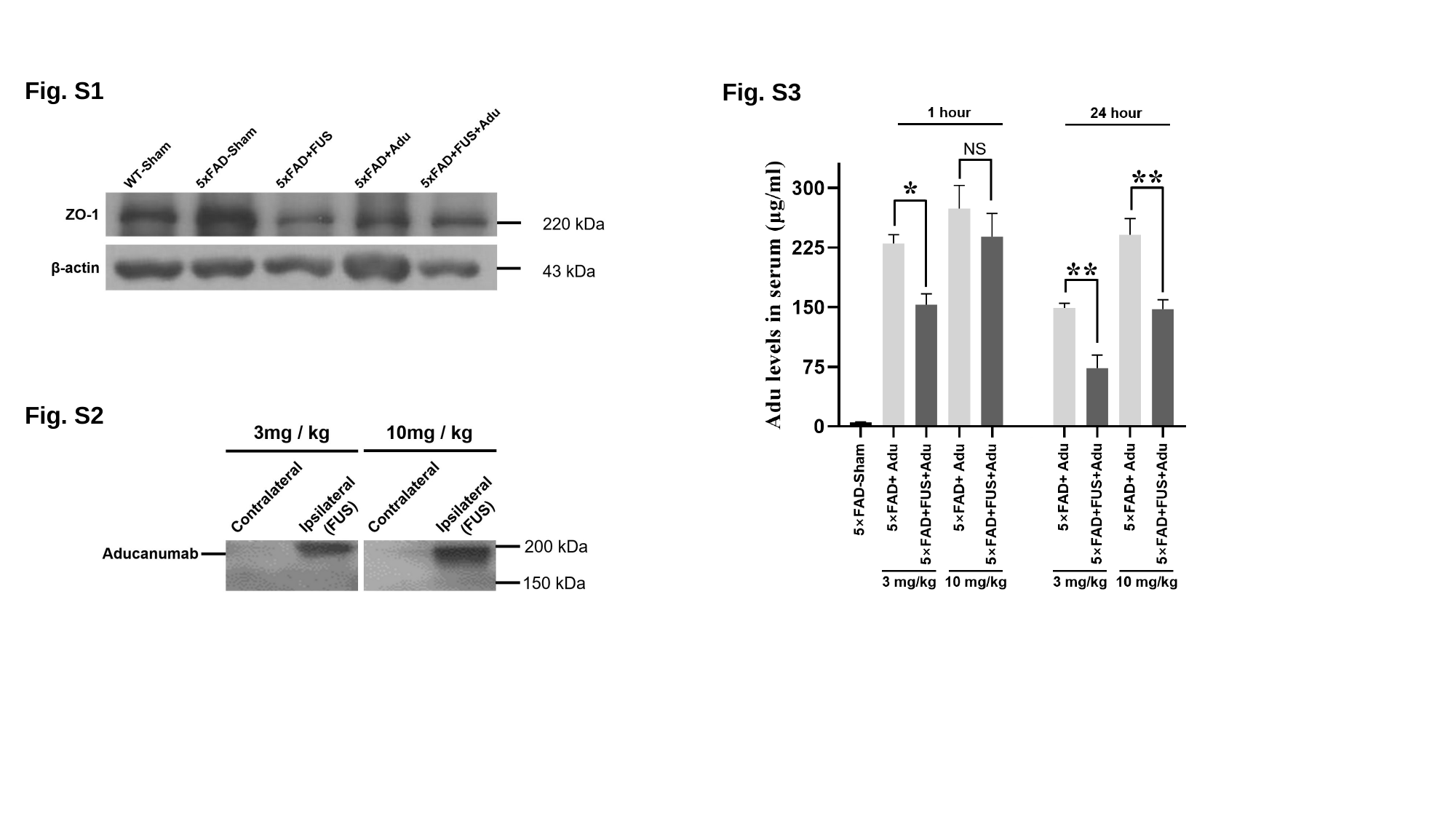

Fig. S1
Fig. S3
Fig. S2

## Slide 2
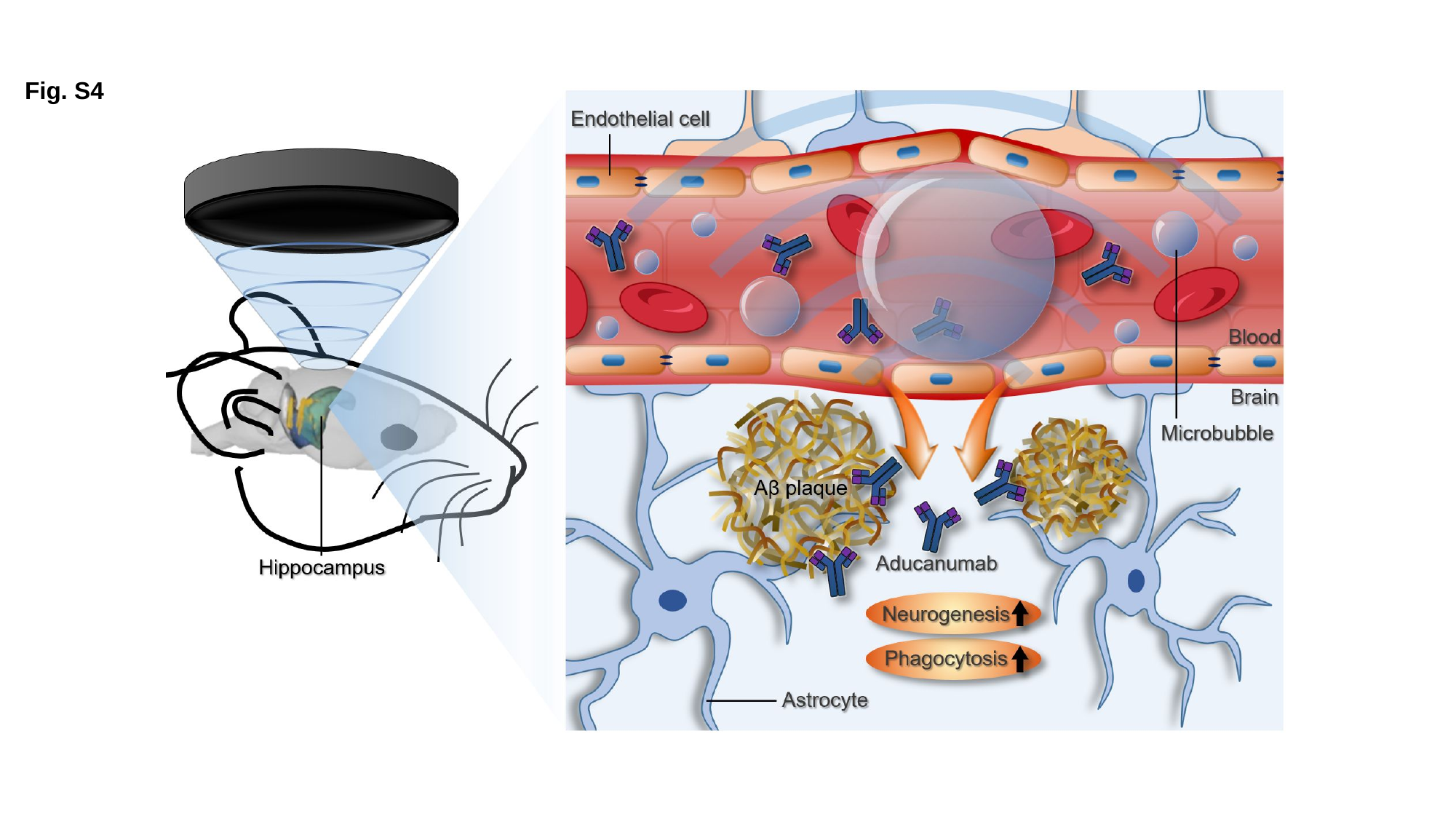

Fig. S4

Supplement: Supplementary file 1 — Additional file 1. Fig. S1: The protein level of ZO-1 in 6-month-old WT-Sham, 5×FAD-Sham, 5×FAD+Adu, 5×FAD+FUS and 5×FAD+FUS+Adu mice. A representative blot of ZO-1 in the hippocampus is shown. Fig. S2: Brain delivery of Adu by FUS occurred in a dose-dependent manner and was examined 24 hours after FUS sonication in 6-month-old 5×FAD mice. A representative blot of Adu assessed with human IgG in the hippocampus is shown. Fig. S3: The Adu concentration in mouse serum after Adu treatment at two time points, 1 hour (white) and 24 hours (gray). A bar graph showing the levels of Adu determined using anti-human IgG antibody in serum. Data are presented as mean ± SEM. Statistical analyses were performed using one-way ANOVA, followed by Tukey’s post-hoc test. (*P < 0.05, **P < 0.01 compared with 5×FAD+Adu mice, n = 3 for each group). Fig. S4: Summary figure. Schematic diagram of the combined therapy of FUS and Adu. FUS can be used in conjunction with microbubbles to temporarily open the BBB, thereby allowing delivery of Adu into the brain. Due to the combined effect of FUS and Adu, neurogenesis and phagocytosis are increased and amyloid plaques are decreased. [file 40035_2022_333_MOESM1_ESM.pptx]
